# Supplementary material for: Chromothripsis during telomere crisis is independent of NHEJ, and consistent with a replicative origin
Source: Genome Res. 2019 May;29(5):737–49. doi: 10.1101/gr.240705.118 (PMC6499312; doi:10.1101/gr.240705.118)
Supplement: Supplemental Material [file supp_gr.240705.118_Supplemental_file_1.zip › contigs/annotated_contigs/DB113/contig.3.DB113_length_473_mean_cov_8.42917547569.docx]

**DB113_length_473_mean_cov_8.42917547569**

```
AAATACAAAAAATTAGCTGGGCGTGGTAGTGTATGCCTGTAGTCCCAGCTACCTGGGAGGCTGAGGCAGGAGAATTGCTTGGGCCCCGG
 >chr10:13279286-13279459 + E=2e-89
AGGCAGAAGTTGCAGTGAACCGAGATCATGCCACTGCACTGCAGCTTGGGCTACAGAGTGAGACTCCATCTAAAAAAAAAAAAA|TTGG

CAG|TTTGTTGTTGTTGTTTGTTTGTTTGTTTTTGTTTTTGTTTTTTTTTT|TG|AGACCCTGTCTCAAAAAAAAAAATCCACAACAAA
    >chr10:12359174-12359221 - E=1e-16               >chr10:13267977-13268225 - E=3e-1
AATAAAAAACTGAGGCTTAGAAAGGATACATGTATACTCAAGGAGACAAGGCTAGTAGATAGCCCTAGCATTAGAACCCCAAGGCTTAT
26
TTGCCAGCAAAGCTCCTGTTCTTGTAAATCAAGGATGAGCAAATGCATAGAGGTCTTGGAGAGCTTCTTTGATCCAGGAATTGGCCCCC

GGATGGAGTGAGTGAGAAAGATAAAGCAGAGT
```
